# Review

**Published:** 1841

**Authors:** 


*Traité des Dents, Manière de diriger la deuxième dentition des enfans, &c. Par C. A. Jamet, Brêvete, Professeur de Prothèsis dentaire, Medicine, Chirurgien-Dentiste de la Faculté de Medicine de Paris, &c. &c. &c — Paris, Cher L' Auteur, rue Notre-Dame de Nazareth, No.* 20, pp. 143, 1839.

The design of the author of the work bearing the above title, seems rather to have been the instruction of parents, and those having the care of children, in the means necessary to the preservation of their teeth, than the practitioner in the treatment of their diseases. To those, therefore, for whom it is intended, it contains, among many erroneous and false notions, as it regards the causes and nature of the maladies of the mouth, much excellent and valuable advice. Some of his remarks on dental hygeia are good ; but his views in regard to the effects produced upon the teeth by the atmosphere and the different winds, which, though held in common with many able and distinguished writers, are altogether visionary and absurd. His observations on caries are ingenious. He says the disease is " produced by ulceration and suppuration of their tissue," (meaning that of the teeth) acknowledging, however, at the same time his ignorance of its causes. He divides the disease into seven species: 1st. Carie Calcarie. 2d. Carie Ecorcante. 3d. Carie Perforante. 4th. Carie Charbonne. 5th. Carie Diruptive. 6th. Carie Stationnaire; and, 7th. Carie Simultant.

Many of the directions which he has given in relation to the management of first and second dentition, are very good; and if every family had a work similar to this, and would be governed by the rules laid down in it, in their attention to the teeth and general constitutional health of their children, a vast amount of pain and suffering would be prevented. But as another French writer has justly remarked, most persons are of opinion that the teeth require no attention, until after the age of the fourteenth or fifteenth year, when, in many instances, disease has extended its desolating ravages so far as to bid defiance to the skill of the dentist.

BALT Ed.

